# Role of Vitamin A in the Immune System

**DOI:** 10.3390/jcm7090258

**Published:** 2018-09-06

**Authors:** Zhiyi Huang, Yu Liu, Guangying Qi, David Brand, Song Guo Zheng

**Affiliations:** 1 Department of Pathology and Physiopathology, Guilin Medical University, Guilin 541004, Guangxi, China; hzy19881021@163.com (Z.H.); liuyu@webmail.hzau.edu.cn (Y.L.); qgy@glmc.edu.cn (G.Q.); 2 Laboratory of Tumor Immunology and Microenvironmental Regulation, Guilin Medical University, Guilin 541004, Guangxi, China; 3 Research Service, VA Medical Center, Memphis, TN 38104, USA; dbrand@uthsc.edu; 4 Department of Medicine, Division of Rheumatology, Milton S. Hershey Medical Center at Penn State University, Hershey, PA 17033, USA

**Keywords:** vitamin A, immunology, infectious disease

## Abstract

Vitamin A (VitA) is a micronutrient that is crucial for maintaining vision, promoting growth and development, and protecting epithelium and mucus integrity in the body. VitA is known as an anti-inflammation vitamin because of its critical role in enhancing immune function. VitA is involved in the development of the immune system and plays regulatory roles in cellular immune responses and humoral immune processes. VitA has demonstrated a therapeutic effect in the treatment of various infectious diseases. To better understand the relationship between nutrition and the immune system, the authors review recent literature about VitA in immunity research and briefly introduce the clinical application of VitA in the treatment of several infectious diseases.

## 1. Introduction

**Figure 1:**
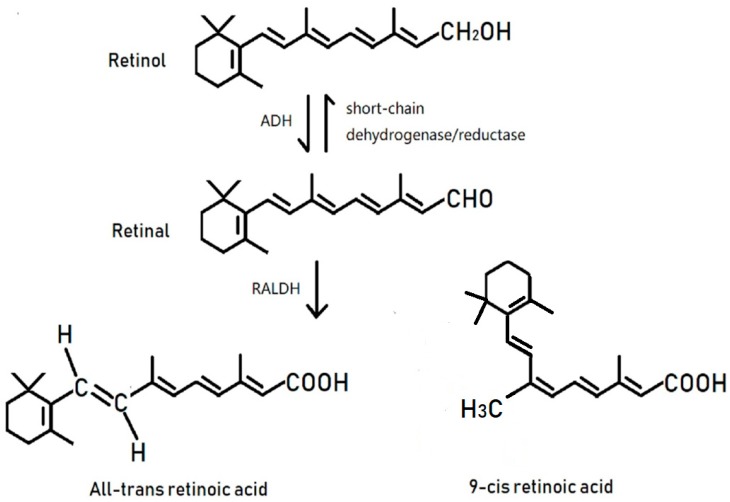
Transformation of retinol into bioactive retinoic acid involves a two-step oxidative reaction. To do this, a group of enzymes, divided in three families, will act together to form the final compound retinoic acid (RA). Retinol transforms into retinal under the catalytic action of the alcohol dehydrogenase (ADH) family; this step can also be regulated by the short-chain dehydrogenase/reductase family, which shows a wide affinity for alcohols and aldehydes. The aldehyde dehydrogenase (RALDH) family then catalyzes retinal to form retinoic acid. Both of the oxidation reactions transmit electrons through the electron acceptor NAD or NADP.

Vitamin A (VitA) is a group of unsaturated monohydric alcohols that contain an alicyclic ring. VitA is insoluble in water but is fat soluble [1]. In 1928, Green and Mellandy reported that VitA could enhance the anti-inflammatory response of organisms and called VitA the “anti-inflammation vitamin” [2]. Later, the anti-inflammatory capacity of VitA was widely studied in the 1980s and 1990s [3,4,5]. VitA exists in the form of retinol, retinal, and retinoic acid (RA), among which RA shows the most biological activity. RA exists in two significant derivatives: 9-*cis*-RA and all-*trans*-RA (ATRA) [6] (Figure 1). The primary biological functions of VitA include maintenance of vision, growth, and the integrity of epithelial and mucous tissue [7]. However, the immunoregulatory mechanisms of VitA are not entirely understood. The authors, here, conduct a detailed review on the most recent advances of VitA function in immunology. We briefly introduce the clinical application of VitA in the treatment of several contagious diseases to provide theoretical support for VitA research in immunology and its therapeutic applications.

## 2. RA Nuclear Receptors

RA is the ligand of the nuclear retinoic acid receptor (RAR) protein. RAR family has three main members (α(isoforms a1-2), β(isoforms b1-4), and γ), which have additional subtypes produced by the use and splicing of alternating promoters [8]. The nuclear RAR acts as a ligand-activating transcription factor, regulating gene transcription according to cell type and tissue [9]. The ATRA is the highest affinity endogenous ligand of RAR [10]. A member of the second protein family, RA-X receptor (RXR) heterodimers and RAR, give high affinity to binding DNA. The RXR family also contains three members (RXRα, RXRβ, and RXRγ). In addition to targeting RARα, RARβ, and RARγ-like ATRA, 9-*cis*-RA also activates RXRα, RXRβ, and RXRγ [11]. RAR/RXR heterologous two dimer-bound DNA is known as the retinoic acid reaction element. The consensus retinoic acid reaction element is composed of two direct repeats of PuG (G/T) and TCA that are most often separated by 5 bases [12]. RAR acts as an enhancer, and promotes chromatin opening and changes in the transcriptional activity of RA target genes when occupied by RA/RAR/RXR complexes [13,14]. Binding of RA to RAR leads to release of the corepressor complex and association with coactivator proteins, followed by altered transcription of downstream target genes and, ultimately, changes in cellular function. RA also undergoes further oxidation by the cytochrome P450 (CYP26) family to more polar metabolites. The lipophilic molecule, RA, can act within the same cell in which it is synthesized (autocrine), or can act in a paracrine manner in nearby cells [15,16,17].

## 3. VitA Is Involved in the Formation of the Epithelial and Mucous Tissues

The epithelium lines all outer surface and most inner surfaces of organisms, and it functions as the “front line” of defense against pathogen invasion. Studies from recent years have shown that VitA plays a crucial role in the morphological formation of the epithelium, epithelial keratinization, stratification, differentiation, and functional maturation of epithelial cells [18]. As a promotor for morphology and a cell differentiation enhancer, VitA is an integral part of the mucus layer of both the respiratory tract and the intestine. Since VitA promotes mucin secretion, it improves the antigen non-specific immunity function of these tissues [18,19]. Research has shown that VitA improves the mechanistic defense of the oral mucosa, increases the integrity of intestinal mucus, and maintains the morphology and amount of urothelium cells [18,19,20].

Even as early as 1925, Wolbach and Howe reported that various epithelia are replaced by stratified squamous keratinizing epithelium when deprived of VitA [21]. It is now clear that under conditions of VitA deficiency (VitAD), epithelial cells shrink, and squamous keratinization may occur in skin, digestive tract, respiratory tract, genitourinary system, cornea, and surrounding soft tissues, leading to symptoms of dry skin, diarrhea, coughing, keratomalacia, corneal opacity, dry eye, and urinary lithiasis [22,23,24,25]. Simultaneously, the resistance of keratinized epithelial tissues to foreign pathogens decreases, and it is no longer able to exert its mechanical barrier function, thus reducing innate immune function and promoting respiratory tract infections, diarrhea, and other diseases in children [26].

## 4. VitA and Its Impact on the Immune System

Immune organs are organs or tissues that realize immune function, and are places where most immunocompetent cells proliferate, differentiate, mature, aggregate, and respond to immunity. Research has shown that crucial immune organs need constant dietary intake to maintain VitA concentrations, and RA was previously shown both to promote the proliferation and to regulate the apoptosis of thymocytes [27,28,29]. In the thymus, endogenous retinoid synthesis and retinoids similar to glucocorticoids might, indeed, be involved in the regulation of thymic proliferation and selection processes, by being present in the thymus in functionally effective amounts [28]. In mice, VitAD leads to a defect in both T cell-mediated and antibody-dependent immune responses [30,31]. VitAD can also inhibit the normal apoptosis process of bone marrow cells, which leads to an increased number of myeloid cells in the bone marrow, spleen, and peripheral blood, indicating that VitA is involved in the regulation of homeostasis of bone marrow [29]. VitA likely regulates the bone marrow population through binding retinoic acid receptor (RAR) in the bone marrow cell nucleus. This binding alters the expression level of apoptosis genes, such as *Bcl-2*, *Fas*, and others. The specific mechanisms by which these apoptosis genes regulate bone marrow homeostasis require further investigation.

## 5. VitA Affects Cell Differentiation, Maturity, and Immunological Function in Innate Immunity

Retinoid acid plays crucial roles in the regulation of the differentiation, maturation, and function of cells of the innate immune system. Innate immune cells are comprised of macrophages and neutrophils, which initiate immediate responses to pathogen invasion through phagocytosis and activation of natural killer T cells which perform immunoregulatory functions through cytotoxic activity [32,33]. There is a report that shows that VitA is essential for the proper development and differentiation of colonic CD169+ macrophages [34]. Macrophages mainly include M1 macrophages secreting proinflammatory cytokines and M2 macrophages expressing anti-inflammatory factors. ATRA inhibits inflammatory reactions by inducing monocyte differentiation toward the macrophage lineage while inhibiting the release of an inflammatory factors from macrophages, thus inducing M1 macrophages in the bone marrow to transform into M2 macrophages [35,36]. ATRA acts on RAR in the nucleus of neutrophils, inducing neutrophil differentiation and heterogeneity through activation of the mTOR signaling pathway. This pathway enhances neutrophil extracellular traps and cytotoxicity, allowing for efficient killing of multiple tumor cells [37]. By downregulating the expression level of IFN-γ and upregulating the secretion of IL-5, RA plays a regulatory role in the early differentiation stage of natural killer T cells [32].

Dendritic cells (DCs) are potent and versatile antigen-presenting cells, and they are specialized sentinels of our immune system capable of orchestrating the innate and adaptive immune response [38]. ATRA can regulate the differentiation of DC precursors [39,40,41]. Bone marrow resident pre-DCs have the potential to differentiate into pre-mucosal DCs (pre-μDCs), characterized by the expression of gut-homing receptors. ATRA acts cell-intrinsically in developing gut-tropic pre-μDCs to effect the differentiation and drive the specialization of intestinal CD103+ DCs [42]. Pre-DCs can migrate to the spleen, where they may sense ATRA skewing the differentiation toward CD11b+CD8− DCs instead of CD11b−CD8α+ DCs [40]. The general consensus on the effect of ATRA on DC function is to promote an anti-inflammatory phenotype characteristic of intestinal DCs [43,44]. However, in the presence of IL-15, ATRA was shown to act as an adjuvant in promoting the secretion of the pro-inflammatory cytokines IL-12 and IL-23 by DCs [45], and has unforeseen co-adjuvant properties that induce Th1 immunity to fed antigens. This suggests that under infectious conditions associated with induction of IL-15 and IL-6 in the intestinal mucosa, ATRA will also promote Th17 immunity [46]. These observations caution against the use of VitA and ATRA for the treatment of autoimmunity and inflammatory intestinal disorders associated with high levels of IL-15.

Innate lymphoid cells (ILC) are a subset of lymphocytes different from T and B cells. Located on the surface of intestinal mucosa, ILCs enhance immune response, maintain mucosal integrity, and promote lymphoid organ formation. ILC can be divided into three groups: ILC1, ILC2, and ILC3. ILC3 are characterized by the expression of the transcription factor RORγt and the cytokines IL-22 and IL-17 [47]. In the fetal period, secondary lymphoid organs formation depends on a subset of ILC3 named lymphoid tissue inducer (LTi) cells [48,49]. Fetal ILC3s are controlled by cell-autonomous RA signaling in utero, which pre-sets the immune fitness in adulthood. Embryonic lymphoid organs contain ILC progenitors that differentiate locally into mature LTi cells. Local LTi was controlled by maternal retinoid intake and fetal RA signaling acting in a hematopoietic cell-autonomous manner. RA controlled LTi cell maturation upstream of the transcription factor RORγt [50]. Both IL-22 and IL-17 mediate antibacterial immune responses and prevent bacterial translocation across barriers. Aberrant regulation of ILC3 and, in particular, the expression of IL-17 is a potential driver of chronic gastrointestinal inflammation [51,52]. Animals deficient in VitA display reduced numbers of ILC3 in contrast to mice fed VitA. This reduction in ILC3 has functional consequences for intestinal immunity, as these mice are more susceptible to infection with the bacterial pathogen *Citrobacter rodentium* than are VitA competent animals [53]. This is primarily due to a lack of ILC3-mediated IL-22 [51,52,53]. RA significantly enhanced IL-22 production by γδ T cells stimulated in vitro with IL-1β or IL-18 and IL-23. In vivo RA shapes early intestinal immune responses by promoting IL-22 synthesis by γδ T cells and ILC [54].

## 6. Effects of VitA on T Cells

### 6.1. RA Induces T Cell Migration

T cells originate from pluripotent stem cells in the bone marrow. These T cells migrate to the thymus where they develop into mature T cells and move to targeted peripheral lymphoid tissues. The entire T cell developmental process is based on the interaction of T cell homing receptors with endothelial adhesion molecules [55]. T cell homing is under the regulation of various adhesion molecules that interact with the homing receptor [55,56,57]. Research has shown that under inflammatory conditions, integrin α4β7 and the T cell chemokine receptor, CCR9, are crucial for T cell migration to the intestine [55,58]. After receiving a RA signal, RARα binds to the RA response element in the integrin α4 gene and regulates the expression of α4β7. Simultaneously, the heterodimer of RARα with the RXR binds to the RAR response element in the promoter region of the CCR9 gene, thus playing an additional regulatory role [59,60,61]. In the intestinal lamina propria, RA is an essential regulator for intestinal homing of CD4+ and CD8+ T cells. VitAD caused a reduction in α4β7(+) memory/activated T cells in lymphoid organs, and a lack of T cells from the intestinal lamina propria [56,57]. Based on this, the provision of ATRA during vaccination can augment the ability of T cell-based viral vaccines to promote the gut/mucosal homing of CD8+ T cells, in order to provide increased protection from mucosal viral challenge, and it also resulted in the formation of more vaccine-specific central memory-like CD8+ T cells in systemic sites [62,63]. Further research shows that RA signaling is required for CD8+ T cells survival and expansion in vivo, and the essential requirement is RARα, but not RARβ or RARγ, for CD8+ T cell survival [64,65]. Whole body imaging using a mouse model of rheumatoid arthritis demonstrated that RA signaling is initiated during the development of inflammation. Furthermore, RA signaling is restricted to the site of inflammation both temporally and spatially. Conditional ablation of RA signaling in T cells significantly interferes with CD4+ T cell effector function, migration, and polarity, indicating RA involvement in T cell migration toward the area of inflammation [66].

### 6.2. RA Is a Control Factor for Regulatory T Cells and Maintains Its Homeostasis

Regulatory T cells (Treg) are a subpopulation of T cells that maintain immune tolerance and regulate the autoimmune response [67,68,69,70]. Foxp3 is a transcription factor that is essential for the differentiation and effector function of Tregs [71,72]. In vivo, ATRA is produced mainly from CD103+ DC in the gut [73]. The cytokine-transforming growth factor-β (TGF-β) converts naïve T cells into Tregs that prevent autoimmunity. However, in the presence of interleukin-6 (IL-6), TGF-β has also been found to promote the differentiation of naïve T lymphocytes into proinflammatory IL-17 cytokine-producing Th17 cells, which promote autoimmunity and inflammation. ATRA, as a key regulator of TGF-β-dependent immune responses, is capable of inhibiting the IL-6-driven induction of proinflammatory Th17 cells and promoting anti-inflammatory Treg cell differentiation [74]. ATRA enhances the expression of Foxp3 in the presence of TGF-β, thus inducing the differentiation of naïve T cells into Tregs and inhibiting the expression of IL-17 [44,71,72,75]. ATRA acts on the nuclear RAR by interacting with TGF-β to activate the ERK1/2 signaling pathway and enhance histone modification of the Foxp3 promotor region and conserved non-coding DNA region. Therefore, ATRA helps maintain Foxp3 gene expression, and regulates Treg differentiation and function [75,76]. Apart from inducing the differentiation of Tregs, ATRA has also been reported to maintain both the stability of Tregs and their immunoregulatory function [45,73,77,78]. In vitro experiments have shown that in pro-inflammatory environments, Tregs are unstable, and can be transformed into other inflammatory cells, such as Th17 cells, by cytokines like IL-6 and IL-21, thus advancing the development of inflammation. Conversely, the addition of ATRA inhibits the transformation of Tregs into Th17 or other Th cells, even in the presence of IL-6, thus maintaining the expression of Foxp3 [73,77]. Local injection of Tregs failed to prevent development in a collagen-induced arthritis model, whereas the injection of ATRA-pretreated Tregs successfully inhibited the development of arthritis [77,78]. ATRA also enhanced the stability and functionality of human natural Treg cells under the inflammatory conditions [79]. ATRA prevented the transformation of Tregs to Th17 cells and other inflammatory cells by inhibiting the expression of IL-6R on the cell surface of peripherally induced Tregs. Therefore, ATRA enhanced IL-2 function, an important immunomodulator, and promoted naïve T cell transformation into natural Tregs while inhibiting the IL-6-induced transformation of naïve T cells into Th17 cells [45,73,78]. Additionally, ATRA also has the ability to induce and promote the development and function of human-induced Treg cells [80].

### 6.3. RA May Promote the Ongoing Immune Response

Although most evidence shows that, at pharmacological levels, RA inhibits the development of inflammatory cells and induces or expands Tregs, recent work has suggested that RA may also promote T cell activation and T helper cell responses at minimal levels.

As mentioned above, RA is mainly produced by DC from the gut. Some reports show that RA may also be produced at other sites during an ongoing immune response [66,81,82]. We have discussed that RA signaling is initiated during the development of inflammation. Similarly, there is evidence demonstrating that the RA–RARα signaling axis is essential for adaptive CD4+ T cell immunity as RARα-deficient CD4+ T cells were less efficient than wild-type counterparts in polyclonal activation. Also, in RARα-deficient T cells, the phosphorylation of PLCγ and ERK1/2 was reduced, and manifests impaired Ca^2+^ mobilization and mTOR/AKT activation upon T cell stimulation. Together, RARα may regulate the signaling pathways downstream of T cell receptor engagement [83].

At pharmacological or high doses (10 nM or higher), RA has been proven to inhibit the reaction of Th17 cells and to induce the generation of Tregs [74,84], and high doses of RA can impair the differentiation of human Th17 and Th1 cells in vitro [85]. However, contrary to reports of RA inhibiting Th1 and Th17 responses, some groups reported that RA was beneficial to Th1 and Th17 cell differentiation at low doses. In physiological doses (1 nM), RA promotes Th17 cell differentiation in vitro [86,87]. In addition, under Th1 or Th17 polarization conditions, the RARα-deficient T cells cultured in vitro did not differentiate into Th1 or Th17 cells, supporting the role of RA in the differentiation of Th1 and Th17 cells, and VitAD mice exhibit significant Th1 and Th17 responses in vivo [53,87,88]. All these results have suggested that RA may have a dose differential effect on the differentiation of Th17 cells and Th1 cells [89]. The role of VitA/RA on Tr1 and Tfh cells is unclear, so far, and warrants further study to allow for clarification.

## 7. Effects of VitA on B Cell Function

### 7.1. Effects of VitA on Immunoglobulin Production

Antibody production by B cells is central to humoral homeostatic maintenance. Antibodies represent a specific class of immunoglobulins. Animal experiments have demonstrated that the addition of carotenoid-rich foods to rabbit diets can increase their serum levels of IgG, IgM, and IgA, thereby enhancing humoral immunity [90]. Further studies in rat have revealed the association between a paucity of VitA in the diet and increased number of DCs, in addition to the significantly upregulated expression of IL-12, Toll-like receptor 2, and myeloid differentiation factor MyD88 in the intestinal mucosa. When the levels of secretory IgA decrease, rats display a decreased immune function, suggesting that VitA is involved in the synthesis of immunoglobulins, and has an important influence on humoral immunity [91]. A report shows that RA potently synergized with gut-associated lymphoid tissues DC-derived IL-6 or IL-5 to induce IgA secretion [92]. A knockout study demonstrated that the ablation of RARα reduces IgA expression by B cells expressed in vivo and in vitro. This indicates that RA acts on B cells directly through RARα, which affects the synthesis and secretion of IgA [93]. It is also likely that RA affects Tregs first, and then indirectly modulates B cells, since Tregs have an important role in regulating B cell responses [94].

### 7.2. VitA Regulation of B Cell Activity

Antigen stimulation of immune cells through specific IgE antibodies results in a rapid, specific hypersensitivity response that is involved in most autoimmune conditions [95]. Evidence shows RA has an IgE-repressive activity in vivo. The inhibitory effect of ATRA on IgE mainly downregulates synthesis and secretion of IgE through RARα, and this inhibitory effect depends on IL-10 [96,97,98,99]. Another report shows that exogenous 9-*cis*-RA in the context of an allergic sensitization profoundly modulates an established humoral IgE response, resulting in reduced specific IgE responses and increased specific IgA responses in mice, indicating that RXR-activating retinoids play a major role in the physiological regulation of IgE due to the endogenous synthesis of 9-*cis*-RA [95]. These make VitA a very promising therapy for the treatment of IgE-mediated hypersensitivity disease.

Regulatory B cells (Breg) are a class of B cell subsets with immunomodulatory functions that are involved in the maintenance of immune homeostasis, and play an essential regulatory role in various immunopathological processes [100,101]. RA can induce the differentiation of naïve B cells into Bregs, and stimulate Breg synthesis and the secretion of IL-10 through RARα [102,103,104,105]. By secreting IL-10, Bregs have ameliorative effects on experimental colitis, arthritis, and lupus [98,102,103,104,105]. The mechanism by which VitA regulates Bregs activity and how it improves its immunomodulating function is not yet understood. Further research will be required to elucidate this question, and to determine whether the effects of VitA on Bregs are stable.

## 8. Application of VitA in the Treatment of Infectious Diseases

### 8.1. Tuberculosis

Tuberculosis, which is a chronic infectious disease caused by the bacterium *Mycobacterium tuberculosis*, is a global health concern. In recent years, the therapeutic outcomes of drugs traditionally used for tuberculosis treatment have worsened because of the development of drug resistance. Therefore, different treatment strategies are required.

Epidemiological studies have shown that the healthy population has a significantly higher serum level of VitA than tuberculosis patients [106,107,108]. A longitudinal cohort study of tuberculosis showed that VitA deficiency is dose-dependently correlated to the occurrence of tuberculosis [109]. An in vitro study demonstrated that RA inhibits the growth of *M. tuberculosis* and reduces its survival rate when engulfed by macrophages [110]. For the mechanism of bacteriocidic activity of VitA, Wheelwright et al. found that VitA can induce the expression of NPC2. In NPC2 gene knockout cells, the stimulation of VitA showed no bacteriocidic activity on infected cells. However, the NPC2 gene is commonly known as a regulator of cholesterol transport rather than an immunological regulatory factor. This result can be explained as follows: cholesterol is the nutritional source for tuberculosis bacterial cell walls, whereas NPC2 facilitates the transportation of cholesterol out of lysosomes, therefore depriving tuberculosis bacteria of their nutritional needs. Without the ability of *M. tuberculosis* to generate protective cell walls, lysozyme can then effectively kill this pathogen [111]. This was demonstrated in a mouse model of tuberculosis in which the addition of ATRA significantly improved the efficacy of traditional anti-tuberculosis drugs [112]. However, more research will be required to elucidate the positive effects ofVitA supplements on the treatment of tuberculosis.

### 8.2. Acquired Immune Deficiency Syndrome (AIDS)

AIDS patients are known, in general, to be deficient in many vitamins [113]. Since various vitamins have the potential to enhance the immunity of the organism and because AIDS arises from human immunodeficiency virus infection, oxidative stress is thought to have an important effect on the infection process of HIV virus [114,115]. VitA, VitC, and VitE are all-natural antioxidants, and by inhibiting the oxidative stress of the organism, it is postulated that these vitamins can ameliorate the progression of AIDS.

A previous study has shown that HIV infection reduces an organism′s regulation of oxidative stress. However, an external antioxidant, such as VitA, does not have any compensatory effect on regulating the oxidative stress response [116]. Furthermore, although HIV-infected individuals are deficient in many different vitamins, vitamin supplementation showed no clinically important benefits in people living with HIV [117]. Consistently, VitA does not influence the vertical transmission of HIV from mother to child [118]. Therefore, VitA supplementation does not appear to affect HIV per se, but that does not mean that HIV patients or carriers should reject the supplementation of VitA or any other vitamins. HIV lowers the immune function of the body, making the patients susceptible to infectious diseases, including tuberculosis, malaria, herpes, and others [119,120]. As mentioned above, VitA enhances the immunity of organisms, and it has been reported to reduce the incidence of tuberculosis in HIV patients [119]. Moreover, pregnancy and postpartum supplementation with a multivitamin significantly improved hematologic status among HIV-infected women and their children, and reduced the risk of anemia [120]. Antiretroviral therapy is the most effective treatment regimen for HIV; however, antiretroviral therapy alone is not sufficient to improve micronutrient deficiency. Therefore, it is essential to supplement VitA, other vitamins, and micronutrients during HIV treatment [121].

### 8.3. Infectious Diseases in Children

Infectious diseases in children were once a global threat [122]. Recent research has suggested a close correlation between a deficiency of micronutrients (particularly VitA) and infectious diseases spread through the respiratory and digestive systems in children [26,123,124]. Meanwhile, many infections result in a decrease in systemic VitA levels as a result of infection-induced anorexia and decreased VitA absorption from the intestine [125,126]. VitA may also be lost in substantial amounts in the urine during infection [127]. As mentioned above, VitA plays a crucial role in the establishment and maintenance of the human immune system. More importantly, VitA has demonstrated a therapeutic effect, to some extent, (see Table 1) in diseases transmitted through the respiratory system, such as pneumonia and measles in children, or in contagious digestive diseases in children, such as infantile diarrhea and hand, foot, and mouth disease [128,129,130]. The World Health Organization has suggested that, in less developed countries, a child between 6 months and 5-years-old should be supplemented with high doses of VitA to prevent and cure VitA deficiency-related diseases, and reduce the incidence and mortality rate of these diseases in children [131].

The recommended daily intake of VitA for children is 1665 IU [132]. VitA, as retinol, exceeds 20,000 IU/d in short periods, leading to intoxication and, occasionally, death. VitA intoxication is a generalized syndrome, the signs and symptoms of which include desquamative and edematous dermatitis, bone pain and tenderness, edema of the extremities and face, irritability, hepatocellular dysfunction, and hypercalcemia [133,134,135,136]. Furthermore, inflammation affects retinoid metabolism. Serum retinol may be sequestered in tissues, leading to a reduction in serum retinol levels, which implies that assessing VitA status with the use of serum retinol during inflammation may be problematic [137].

## 9. Summary

As the interdisciplinary approach continues to develop in research, people have been paying increasing attention to the relationship between nutrition and immunity. Furthermore, the influence of micronutrients on the immune function of the organism has been widely studied. VitA has both promoting and regulatory roles in both the innate immune system and adaptive immunity; therefore, it can enhance the organism’s immune function and provide an enhanced defense against multiple infectious diseases. Currently, the VitA’s effect on immune function has been studied at the molecular level, and more research is ongoing about the therapeutic effects of VitA on preventing and curing various infectious diseases. As increasing evidence appears with time, VitA will likely play more critical roles in modern therapeutics.

## Figures and Tables

**Table 1:** The therapeutic effect of VitA on several infantile infectious diseases.

Diseases	Role of VitA	Method Setting	Model [Reference]
Measles	Reduce mortality	Meta-analysis	Human [138]
Measles	Reduce morbidity and mortality	Systematic review and meta-analysis	Human [129]
Measles	Reduce mortality	Meta-analysis	Human [139]
Measles	Reduce morbidity	Randomized double-blind controlled trial	Human [140]
Acute pneumonia	Promoting the production of specific antibodies	Randomized controlled trial	Mice [141]
Acute pneumonia	Relieving clinical symptoms and signs	Meta-analysis	Human [128]
Infantile diarrhea	Reduce morbidity and mortality	Systematic review and meta-analysis	Human [129]
Infantile diarrhea	Promote the production of IgA in the intestinal tract and enhance the mucosal immune function	Randomized controlled trial	Mice [142]
Infantile diarrhea	Reduce morbidity	Randomized double-blind controlled trial	Human [140]
Enteric infection	Reduce morbidity and mortality	Randomized controlled trial	Mice [143]
Malaria	Reduce morbidity	Randomized double-blind controlled trial	Human [144]
Malaria	Reduce morbidity	Randomized controlled trial	Human [145]
Malaria	Reduce morbidity	Randomized double-blind controlled trial	Human [146]
Hand foot and mouth disease	Promote production of immunoglobulin and enhance antiviral function	Cross-sectional observation and study	Human [130]
Mumps	Up-regulation of type 1 interferon and inhibition of viral replication	In vitro controlled experiment	Cells [147]
